# Measurement and Monte Carlo simulation for energy‐ and intensity‐modulated electron radiotherapy delivered by a computer‐controlled electron multileaf collimator

**DOI:** 10.1120/jacmp.v15i1.4506

**Published:** 2014-01-06

**Authors:** Lihui Jin, Ahmed Eldib, Jinsheng Li, Ismail Emam, Jiajin Fan, Lu Wang, C‐M Ma

**Affiliations:** ^1^ Department of Radiation Oncology Fox Chase Cancer Center Philadelphia PA USA; ^2^ Clinical Oncology Department Faculty of Medicine, Ain Shams University Cairo Egypt

**Keywords:** Monte Carlo simulation, modulated electron radiotherapy (MERT), eMLC

## Abstract

The dosimetric advantage of modulated electron radiotherapy (MERT) has been explored by many investigators and is considered to be an advanced radiation therapy technique in the utilization of electrons. A computer‐controlled electron multileaf collimator (MLC) prototype, newly designed to be added onto a Varian linac to deliver MERT, was investigated both experimentally and by Monte Carlo simulations. Four different electron energies, 6, 9, 12, and 15 MeV, were employed for this investigation. To ensure that this device was capable of delivering the electron beams properly, measurements were performed to examine the electron MLC (eMLC) leaf leakage and to determine the appropriate jaw positioning for an eMLC‐shaped field in order to eliminate a secondary radiation peak that could otherwise appear outside of an intended radiation field in the case of inappropriate jaw positioning due to insufficient radiation blockage from the jaws. Phase space data were obtained by Monte Carlo (MC) simulation and recorded at the plane just above the jaws for each of the energies (6, 9, 12, and 15 MeV). As an input source, phase space data were used in MC dose calculations for various sizes of the eMLC shaped field ($10\times 10\;{\text{cm}}^{2}$, $3.4\times 3.4\;{\text{cm}}^{2}$, and $2\times 2\;{\text{cm}}^{2}$) with respect to a water phantom at source‐to‐surface distance (SSD)=94cm, while the jaws, eMLC leaves, and some accessories associated with the eMLC assembly as well were modeled as modifiers in the calculations. The calculated results were then compared with measurements from a water scanning system. The results showed that jaw settings with 5 mm margins beyond the field shaped by the eMLC were appropriate to eliminate the secondary radiation peak while not widening the beam penumbra; the eMLC leaf leakage measurements ranged from 0.3% to 1.8% for different energies based on in‐phantom measurements, which should be quite acceptable for MERT. Comparisons between MC dose calculations and measurements showed agreement within 1%/1mm based on percentage depth doses (PDDs) and off‐axis dose profiles for a range of field sizes for each of the electron energies. Our current work has demonstrated that the eMLC and other relevant components in the linac were correctly modeled and simulated via our in‐house MC codes, and the eMLC is capable of accurately delivering electron beams for various eMLC‐shaped field sizes with appropriate jaw settings. In the next stage, patient‐specific verification with a full MERT plan should be performed.

PACS number: 87.55.ne

## I. INTRODUCTION

For decades, megavoltage electron beams have represented an important treatment modality in modern radiotherapy, providing a unique option in the treatment of malignancies confined to superficial regions (less than 5 cm deep) due to a characteristically sharp dose fall‐off beyond the tumor. However, the unique physical characteristics of the electron beam have been underutilized due to limitations of the current available technology. Energy‐ and intensity‐modulated electron therapy (MERT) for fixed beam treatment has been an active research topic because it may provide more efficient and effective treatment for superficial lesions, greatly enhancing the utilization of electron beams in radiotherapy.^(1)^

MERT is designed to modulate both electron energy and beam intensity to achieve the dose distribution conformal to the tumor target, resulting in greater normal tissue sparing as compared to the conventional approach with an applicator/cutout, usually one static single‐energy field.^(2)^ The dosimetric advantage of using MERT over other electron and photon modalities has been investigated by many groups. Ma et al.^(3)^ published a comparative dosimetric study on tangential photon beams, intensity‐modulated radiation therapy (IMRT), and MERT for breast cancer treatment, showing a reduced maximum dose to the lung and heart, while Xiong et al.^(4)^ from Ma's group later demonstrated that using combined modulated electron and photon therapy for breast cancer may have an advantage over conventional treatment techniques. Al‐Yahya et al.^(5)^ showed the efficacy of MERT in combination with three‐dimensional conformal therapy and IMRT with a reduced whole body dose over photon modalities alone. Recently, additional dosimetric benefits using MERT technology as compared to other modalities, including volumetric‐modulated photon arc therapy, were investigated in treating a postmastectomized chest wall, head and neck shallow tumors, and tumor beds of the breast by various groups.^6^, ^7^, ^8^, ^9^

However, a significant challenge emerged regarding the delivery of MERT because the traditional approach using a conventional treatment head, which employs a cutout (or a block) to shape electron beams and bolus material to modify beam penetration/intensity, was not clinically practical due to the substantial amount of time required to create the beam modifiers and adjust cutouts for the treatment. Various groups have been investigating an efficient and clinically practical means to deliver MERT. Previous research on the electron MLC (eMLC) for MERT delivery is documented in representative literatures by Ma et al.,^(2)^ Gauer et al.,^(10)^ Hogstrom et al.,^(11)^ Al‐Yahya et al.,^(12)^ Vatanen et al.,^(13)^ and Eldib et al.,^(14)^ while efforts on using photon MLC (pMLC) to deliver MERT were also made by many investigators including du Plessis et al.,^(15)^ Klein et al.,^(16)^ Jin et al.,^(17)^ Karlsson et al.,^(18)^ and Mihaljevic et al.^(19)^ Recently, Connell et al.^(20)^ published a study on the use of scattering foil free beams for modulated electron radiotherapy, an experimental feasibility study on the use of scattering foil free beams for modulated electron radiotherapy, exploring the potential benefit of removing the scattering foil from the beam line due to the significant reduction of the bremsstrahlung tail dose.

The calculation of dose distributions for electron beam radiotherapy is also challenging because electron scattering in matter is strongly influenced by density and material composition in patients. As Ma et al.^(21)^ and various investigators including Mackie et al.,^(22)^ Kawrokaw et al.,^(23)^ Mohan,^(24)^ and Kapur^(25)^ pointed out, the current widely adopted 3D pencil beam algorithm^(26)^ by most treatment planning systems has limitations with small irregular electron fields and the presence of inhomogeneities. Jin et al.^(17)^ experimentally verified the accuracy of electron doses calculated from the Monte Carlo method employed in our in‐house inverse planning system, which was used for an inhomogeneous breast phantom‐based plan containing breast and lung tissue geometry involving mixed electron energies (6, 9, 12, and 15 MeV) and 22 different sized photon MLC‐shaped field segments that ranged from about $1\times 1\;{\text{cm}}^{2}$ to $21\;\text{cm}\times 21\;{\text{cm}}^{2}$).

Following previous research results for eMLC,^2^, ^14^ a computer‐controlled prototype (eMLC) has been manufactured and is now available in our institution. The purpose of this work is to investigate this device, assuring it is capable of accurately delivering the MERT. In this work, we experimentally determine appropriate jaw settings for the eMLC‐shaped fields, examine leaf leakage and establish baseline beam data for benchmarking MC calculations while the beam phase space data for 6, 9, 12, and 15 MeV electrons, which are input sources for the MC dose calculation in our in‐house inverse MERT planning system, were acquired by MC beam simulations and commissioned by comparing dose profiles and percentage depth doses between the MC calculations and measurements for energies of various field sizes. Other higher energies (18 and 22 MeV) available in the Varian linac were not considered in our research at this step because it is realized that these high electron energies do not present very fast dose fall‐off after the therapeutic region, and thus utilizing them may compromise the benefit of MERT in reducing doses to distal critical structures.

## II. MATERIALS AND METHODS

### A. The prototype eMLC

**Figure 1:**
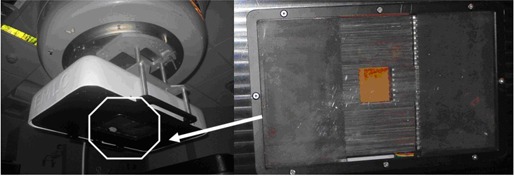
The computer‐controlled eMLC prototype which is an add‐on device for a Varian standard linac.

The eMLC device is an add‐on tool designed to be attached to the Varian linac (Fig. 1) (Varian Medical Systems, Palo Alto, CA), containing 27 pairs of tungsten leaves with a 0.56 cm width and 2 cm thickness, providing a field size as large as $17.6\times 17.6\;{\text{cm}}^{2}$ defined at 100 cm source‐to‐surface distance (SSD). The leaves have a tongue‐and‐groove shape with straight ends, which will ensure minimal interleaf leakage. The distance between the bottom of the eMLC leaves and the linac source is 84 cm, while the bottom of the plastic exterior of the entire eMLC assembly is distanced from the linac source by 87.6 cm. With 94 cm of SSD in the treatment, there is approximately a 6.4 cm air gap for clearance between the eMLC device bottom and the patient surface. The loading of MERT plans, as well as delivery by the eMLC device, can be managed by software which accompanies the device.

### B. Measurements

#### B.1 Definition of field size for the eMLC‐shaped field

For convenience, in this paper, we define the nominal field size of a particular eMLC‐shaped field as its light field size (i.e., the projected size of the light field as defined by the eMLC opening at a 100 cm SSD). It should be noted, for an eMLC‐shaped electron field, that the nominal field size does not necessarily coincide with the electron radiation field size defined by FWHM of the dose profile because the light field size that is used for defining the electron nominal field size mentioned above is conventionally calibrated using photon beams by checking the coincidence of the photon radiation field and the light field. Due to the strong scattering properties of the electrons in air and in the linac component, the geometric fall‐off of the electron fluence does not completely follow the inverse square law. Hence, it is not necessarily true for an electron field shaped by the eMLC to be consistent with its light field over a certain range of SSDs (e.g., from 60 cm to 120 cm). In this paper, the nominal size of the eMLC field is simply addressed as the field size, which is also normally defined at SSD=100cm unless specified.

#### B.2 Investigation of appropriate jaw settings

The eMLC was attached to a Varian linac containing upper and lower jaws above the eMLC. In order to deliver MERT, proper positioning of the jaws for each eMLC‐shape field is of paramount importance in eliminating strayed electrons that may cause a secondary dose peak (see Results section 1) without compromising characteristics of off‐axis dose profiles. To investigate this issue, experimental measurements were performed for a small field $\left(2\times 2\;{\text{cm}}^{2}\right)$ with SSD=94cm and a large field $\left(10\times 10\;{\text{cm}}^{2}\right)$ with SSD=100cm with various jaw settings. The measurements were done with films (Kodak EDR2; Eastman Kodak Company, Rochester, NY) in a $30\times 30\times 30\;{\text{cm}}^{3}$ solid water phantom consisting of slabs of various thicknesses. To cover the range of electron energies used in this work for the MERT, both low energy (6 MeV) and high energy (15 MeV) were used for the experimental measurements.

#### B.3 Leaf leakage measurement

To confirm that the eMLC leaf leakage was acceptable, measurements were performed both in air and in a phantom, respectively, for each of the electron energies. The measured leaf leakage is quantified by taking the ratio of the measured doses at the beam axis with all leaves closed and a $10\times 10\;{\text{cm}}^{2}$ eMLC‐shaped field. For the in‐air measurement, the distance between the source and the detector (0.6 cc Farmer chamber, EXRADIN A12; Standard Imaging Inc., Middleton, WI) was 94 cm, while the in‐phantom measurement was performed at the depth of a maximum dose for each of the energies with 94 cm SSD in a solid water phantom with a dimension of $30\times 30\times 30\;{\text{cm}}^{3}$.

In addition, the film measurements were performed with all the eMLC leaves closed at SSD=94cm for 6 MeV and 15 MeV, respectively. The films were placed on the surface of the same solid water as used for the ion chamber measurement.

#### B.4 Measurements of percentage depth doses and off‐axis dose profiles

In order to ensure accurate phase space representation of clinical electron beams for MC dose calculations, the percentage depth doses (PDDs) and off‐axis dose profiles with selected field sizes ($2\times 2\;{\text{cm}}^{2}$, $3.4\times 3.4\;{\text{cm}}^{2}$, and $10\times 10\;{\text{cm}}^{2}$) at the SSD=94cm for electron beam energies of 6, 9, 12, and 15 MeV were measured in water and used to benchmark the MC calculations. The PTW MP3 water scanning system (PTW, Freiburg, Germany) with PTW 0.125 cc ion chambers (cylindrical with 6 mm diameter) was used for the measurements. In the MC dose calculations used for comparison with the measurements, the voxel size chosen was 5 mm to match the ion chamber volume to minimize potential dose differences caused by the spatial averaging effect of the detector.

### C. Monte Carlo beam simulation and dose calculation

The MCBEAM, an in‐house EGS4/PRESTA user code^(27)^ was employed in this work to obtain phase space data for electron beam energies 6, 9, 12, and 15 MeV in a Varian Trilogy linac on which the eMLC is attached. The dimensions and materials for the accelerator components were incorporated into the Monte Carlo simulation according to the manufacturer's specifications. Manufacturer‐supplied energy spectra of electron beams emerging from the vacuum exit window were initially used in the simulation and were then fine‐tuned to achieve agreement between measured depth‐dose curves along the central axis and Monte Carlo dose calculations. Simulated beam phase space data were scored at a plane just above the jaws as an input source of Monte Carlo dose calculation for MERT planning.^(17)^ In the accelerator simulation, the energy cutoffs for electron transport (ECUT and AE) were 700 keV (total energy) and for photon transport (PCUT and AP) 10 keV, respectively. The electron step length was confined such that the maximum fractional energy loss per electron step was 4% (i.e., ESTEPE=0.04). Stopping power values recommended by ICRU for different compositions were used in the simulation.^(28)^ Air in the particle transport path was simulated. The number of particles (including electrons, photons, and positions) in a phase space file ranged up to 100 million.

The in‐house code MCPLAN,^(17)^ an expanded version of MCSIM, an EGS4/PRESTA user code,^(29)^ performs inverse treatment planning of both photon and electron beams with the 3D CT patient data. In this work, MCPLAN used the phase space data as the source input for dose calculations with simulation parameters ECUT, AE, PCUT, AP, as well as ESTEPE, set the same as with the MCBEAM code. The jaws and the eMLC were simulated as beam modifiers in MCPLAN together with a 3D rectilinear patient or phantom. The eMLC was simulated with actual geometric and material parameters, except for the tongue‐and‐groove structure that proved insignificant for electron beams in our previous study.^2^, ^17^ The material and mass densities of the individual voxels were obtained based on CT numbers using a piecewise linear conversion curve.^(21)^ In the dose calculations, the dose uncertainties (one standard deviation) were less than 1% of the maximum dose.

## III. RESULTS & DISCUSSION

### A. Determination of jaw settings

**Figure 2:**
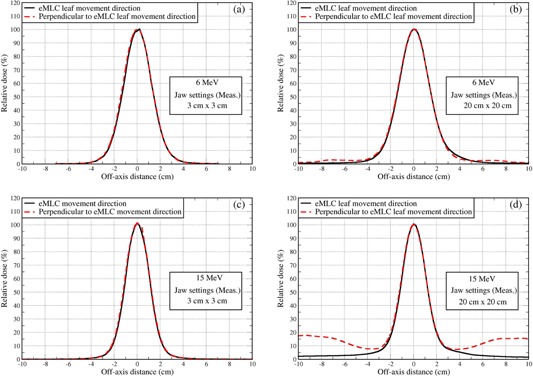
Measured off‐axis dose profiles with SSD=94cm for field size $2\times 2\;{\text{cm}}^{2}$ along directions of the eMLC leaf movement and perpendicular to the eMLC leaf movement, respectively: (a) 6 MeV at the depth of 1.35 cm for jaw settings at $3\times 3\;{\text{cm}}^{2}$; (b) the same as (a), except jaw settings at $20\times 20\;{\text{cm}}^{2}$; (c) the same as (a) except for at a depth of 3.0 cm for the 15 MeV; and (d) the same as (c) except jaw settings at $20\times 20\;{\text{cm}}^{2}$.

Figure 2 showed off‐axis dose profiles of a $2\times 2\;{\text{cm}}^{2}$ field shaped by eMLC at depths of maximum doses (1.5 cm for 6 MeV and 3.0 cm for 15 MeV) with jaw settings at $3\times 3\;{\text{cm}}^{2}$ and $20\times 20\;{\text{cm}}^{2}$, respectively. From the figure, when the jaws are set at $20\times 20\;{\text{cm}}^{2}$, secondary dose peaks can be seen outside the defined radiation field in the direction perpendicular to the eMLC leaf movement for both energies, but much more pronounced for 15 MeV. On the other hand, the dose profiles for the eMLC leaf movement direction present no secondary dose peaks and do not noticeably vary with jaw settings. It was found that for the direction perpendicular to the eMLC leaf movement, the jaw settings at $3\times 3\;{\text{cm}}^{2}$ for the radiation field $2\times 2\;{\text{cm}}^{2}$ were small enough to eliminate the secondary dose peak for both energies, producing the same off‐dose profiles as in the eMLC movement direction (Fig. 2). In other words, setting the jaw with a 5 mm margin beyond the field size for the small field can eliminate secondary dose peaks in the direction perpendicular to the eMLC leaf movement without compromising the characteristics of dose profiles in the field region including penumbra.

Secondary dose peaks occur in the direction perpendicular to the eMLC leaf movement when using larger jaw settings because there is a smaller dimension in this direction occupied by eMLC leaves and, thus, more scattered or even primary electrons can leak into the peripheral region from this direction.

**Figure 3:**
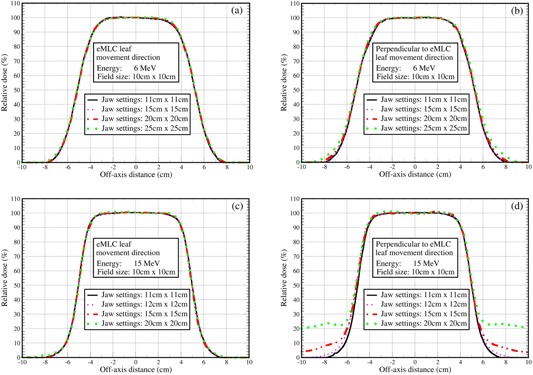
Measured off‐axis dose profiles for the field size $10\times 10\;{\text{cm}}^{2}$ with SSD=100cm and the jaw settings as indicated in the plot: (a) 6 MeV at the depth of 1.35 cm along the direction of the eMLC leaf movement; (b) the same as (a) except for the direction perpendicular to the eMLC leaf movement; (c) the same as (a) except for at a depth of 3.0 cm for the 15 MeV; and (d) the same as (c) except for the direction perpendicular to the eMLC leaf movement.

The experimental measurements performed for a larger field size, $10\times 10\;{\text{cm}}^{2}$, demonstrated similar results as those for the $2\times 2\;{\text{cm}}^{2}$ field size. For the eMLC leaf movement direction, the dose profiles again do not change noticeably with jaw settings, while the dose profiles in the direction perpendicular to the eMLC leaf movement exhibited reduced radiation leakage outside the field as the jaws were positioned closer to the edge of the field shaped by the eMLC (Fig. 3). In Fig. 3, the results for four different jaw settings ($11\times 11,15\times 15,20\times 20,25\times 25\;{\text{cm}}^{2}$ jaw settings for 6 MeV, and $11\times 11,12\times 12,15\times 15,20\times 20\;{\text{cm}}^{2}$ jaw settings for 15 MeV), were plotted. When jaws were set to $11\times 11\;{\text{cm}}^{2}$, dose profiles showed no visible differences between both eMLC leaf movement and its perpendicular direction, clearly demonstrating that setting the jaw with a 5 mm margin beyond the field shaped by the eMLC is also appropriate for the large field size in order to remove the secondary dose peaks while no characteristics of dose profiles are compromised.

### B. Leaf leakage measurement

**Figure 4:**
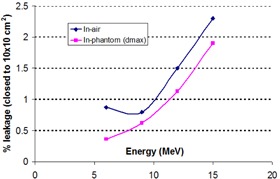
Measured eMLC leaf leakage vs. electron beam energy. The measured leaf leakage is quantified by taking the ratio of measured doses at beam axis with all leaves closed and an open $10\times 10\;{\text{cm}}^{2}$ eMLC‐shaped field. For the in‐air measurement, the distance between the source and the detector (0.6 cc Farmer chamber) is 94 cm, while the in‐phantom measurement was performed at a depth of maximum dose for each of the energies with SSD=94cm in a solid water phantom measuring $30\times 30\times 30\;{\text{cm}}^{3}$.

The measured results in air and in phantom were plotted in Fig. 4, showing: 1) leaf leakage generally increases with energy; 2) in‐phantom measurements present smaller leaf leakage than in‐air measurements; and 3) the maximum leakage that occurs at 15 MeV is less than 2.5% for in‐air measurements and less than 2.0% for in‐phantom measurements. Since scattered electrons and bremsstrahlung from leaf radiation leakage for a higher energy electron beam are capable of penetrating longer distances and more of them are able to reach the detector, the leaf radiation leakage should be expected to increase with energy. The in‐phantom measured leakage is lower than the in‐air measured leakage, possibly because most radiation leakage contains lower energy electrons, which will lead to fewer electrons having the capability of reaching the detector in the depth of the phantom as compared to the in‐air measurement. In the MERT planning, the eMLC leakage effects on doses are accounted by simulating eMLC and jaws as modifiers in our planning system.^(17)^

In addition, film measurements showed the flat dose profile as expected, which can be explained by the tongue‐and‐groove design of the eMLC leaf and scatter characteristics of electrons in air, suggesting the dose measured at any point in the file includes contribution from both interleaf and intraleaf leakage.

### C. Measurements and Monte Carlo simulations

**Figure 5:**
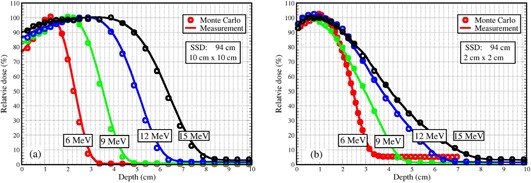
Comparison between Monte Carlo simulated and measured percentage depth doses along the beam axis for 6, 9, 12, and 15 MeV, respectively, with the eMLC‐shaped fields of (a) $10\times 10\;{\text{cm}}^{2}$ and (b) $2\times 2\;{\text{cm}}^{2}$ at SSD=94cm.

**Figure 6:**
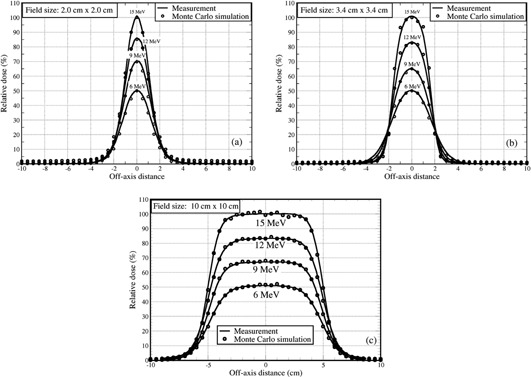
Comparison between Monte Carlo simulated and measured off‐axis dose profiles at SSD=94cm for: (a) the eMLC shaped field of $2\times 2\;{\text{cm}}^{2}$ at a depth of 1.3, 2.15, 2.8, and 3.3 cm, respectively, for 6, 9, 12, and 15 MeV electron beams; (b) $3.4\times 3.4\;{\text{cm}}^{2}$ at the depth of 1.35 cm for the same four energies; and (c) $10\times 10\;{\text{cm}}^{2}$ at a depth of 1.0, 1.8, 2.75, and 3.0 cm, respectively, for 6, 9, 12, and 15 MeV.

Linac components, including exit window, primary and secondary scattering foils, and monitor unit chambers were included in MC beam simulation using the code MCBEAM to acquire phase space data for each of the energies, which were scored below the monitor unit chambers but above the jaws of the linac. For dose calculations, the MCPLAN simulated the jaws, eMLC leaves, and other eMLC accessories as modifiers, together with a 3D rectilinear patient or phantom. The measured PDDs with $10\times 10\;{\text{cm}}^{2}$ and $2\times 2\;{\text{cm}}^{2}$ fields shaped by eMLC for different electron energies were plotted along with the MC calculated PDDs (Fig. 5), showing the agreement to be within 1%/1mm. Off‐axis dose profiles for different field sizes ($2\times 2\;{\text{cm}}^{2}$, $3.4\times 3.4\;{\text{cm}}^{2}$, and $10\times 10\;{\text{cm}}^{2}$) at depths were compared between measurements and MC calculations, as shown in Fig. 6, in which a 1%/1mm agreement was achieved.

## IV. CONCLUSIONS

This paper presents our work with respect to a motorized eMLC prototype, which is designed to deliver MERT in a Varian linac machine.

Proper jaw settings have been determined by a series of measurements for eliminating the strayed radiation outside the field penumbra region. In addition, we assessed the eMLC leakage by measurement both in air and in phantom. We concluded that when using the eMLC device to deliver MERT, the jaw settings with a 5 mm margin beyond the field shaped by the eMLC were appropriate to eliminate the secondary radiation peak while not widening the beam penumbra; the measured eMLC leaf leakage for the in‐phantom measurement ranged from 0.3% to 1.8% for various energies and should be acceptable for MERT. As previously discussed in the Introduction section, the MC dose algorithm is needed for MERT to achieve the required dose accuracy. It is, therefore, important to verify that the eMLC can accurately deliver doses as calculated using the MC method employed in our in‐house planning system. In the dose calculations, jaws, eMLC leaves, and other assembly components were simulated as modifiers in our planning system. The results showed agreement within 1%/1mm between MC dose calculations and measurements based on PDDs and off‐dose profiles for a range of field sizes for each of the electron energies. This indicated that the eMLC, as well as other components in the electron beam path, had been properly modeled in MC beam simulation/dose calculations.

Our current work has demonstrated that the eMLC is capable of accurately delivering electron beams with various sized fields with appropriate jaw settings. Future investigations should include patient‐specific verification of MERT planning.

## ACKNOWLEDGMENTS

We would like to thank Lorraine Medoro for her assistance in editing and proof reading this manuscript.

## Supporting information

Supplementary Material
